# Extracellular vesicles in hematological malignancies: EV-dence for reshaping the tumoral microenvironment

**DOI:** 10.3389/fimmu.2023.1265969

**Published:** 2023-09-26

**Authors:** David Van Morckhoven, Nathan Dubois, Dominique Bron, Nathalie Meuleman, Laurence Lagneaux, Basile Stamatopoulos

**Affiliations:** ^1^ Laboratory of Clinical Cell Therapy, Jules Bordet Institute, Université Libre de Bruxelles (ULB), Brussels, Belgium; ^2^ Departement of Hematology, Jules Bordet Institute, Brussels, Belgium

**Keywords:** extracellular vesicles, exosomes, microparticles, hematological malignancies, microenvironment, cellular communication, leukemia, multiple myeloma

## Abstract

Following their discovery at the end of the 20th century, extracellular vesicles (EVs) ranging from 50-1,000 nm have proven to be paramount in the progression of many cancers, including hematological malignancies. EVs are a heterogeneous group of cell-derived membranous structures that include small EVs (commonly called exosomes) and large EVs (microparticles). They have been demonstrated to participate in multiple physiological and pathological processes by allowing exchange of biological material (including among others proteins, DNA and RNA) between cells. They are therefore a crucial way of intercellular communication. In this context, malignant cells can release these extracellular vesicles that can influence their microenvironment, induce the formation of a tumorigenic niche, and prepare and establish distant niches facilitating metastasis by significantly impacting the phenotypes of surrounding cells and turning them toward supportive roles. In addition, EVs are also able to manipulate the immune response and to establish an immunosuppressive microenvironment. This in turn allows for ideal conditions for heightened chemoresistance and increased disease burden. Here, we review the latest findings and reports studying the effects and therapeutic potential of extracellular vesicles in various hematological malignancies. The study of extracellular vesicles remains in its infancy; however, rapid advances in the analysis of these vesicles in the context of disease allow us to envision prospects to improve the detection and treatment of hematological malignancies.

## Introduction

The interaction between neoplastic cells and the microenvironment (ME) is one of the main drivers in the progression of several hematological malignancies (HMs). Malignant cells interact with the ME, impacting resistance to apoptosis, survival, proliferation, migration, and chemoresistance (1). Furthermore, this crosstalk allows malignant cells to manipulate the immune response by hindering immune surveillance and fostering an immunosuppressive ME, allowing the disease to progress (2, 3). This interaction occurs in different ways: direct cell-to-cell contact, secretion of soluble factors, or release of extracellular vesicles (EVs) (1). Recently, EVs have increasingly proven to be of interest in HM research. An introduction to the impact of EVs on the signaling networks of leukemias and multiple myeloma as well as their potential therapeutic applications will be discussed in this review.

## Extracellular vesicles: definition, biogenesis and cargo

First thought to be “platelet dusts”, cell debris or experimental artifacts in the second half of the 20^th^ century (4), little interest was originally given to EVs. In 1996, Raposo and colleagues demonstrated the ability of EVs to act as antigen-presenting entities, and their biological significance was highlighted for the first time (5). Currently, EVs have been acknowledged as one of the main intercellular communication drivers (6). Their relevance for the progression of HMs cannot be ignored, as they shape the ME responsible for nurturing neoplastic cells, thus driving malignancy (6). EVs commonly referred to as ‘exosomes’ (30-150 nm in diameter) and ‘microparticles’ (100-1,000 nm in diameter) will be referred to as small EVs (sEVs, <150 nm in diameter) and medium/large EVs (m/lEVs, >150 nm in diameter), respectively, when mentioned. The different types of EVs are distinguished by their biogenesis. sEVs mainly arise from the endosomal compartment, whereas m/lEVs arise from plasma membrane budding (7, 8). Additionally, ‘apoptotic bodies’ (>1,000 nm in diameter) emerge during cell apoptosis and exhibit typical characteristics of cell death (condensed chromatin, fragmented DNA and proteolytic cleavage of the cytoskeleton). We will mainly focus our attention on sEVs and m/lEVs.

**Figure 1:**
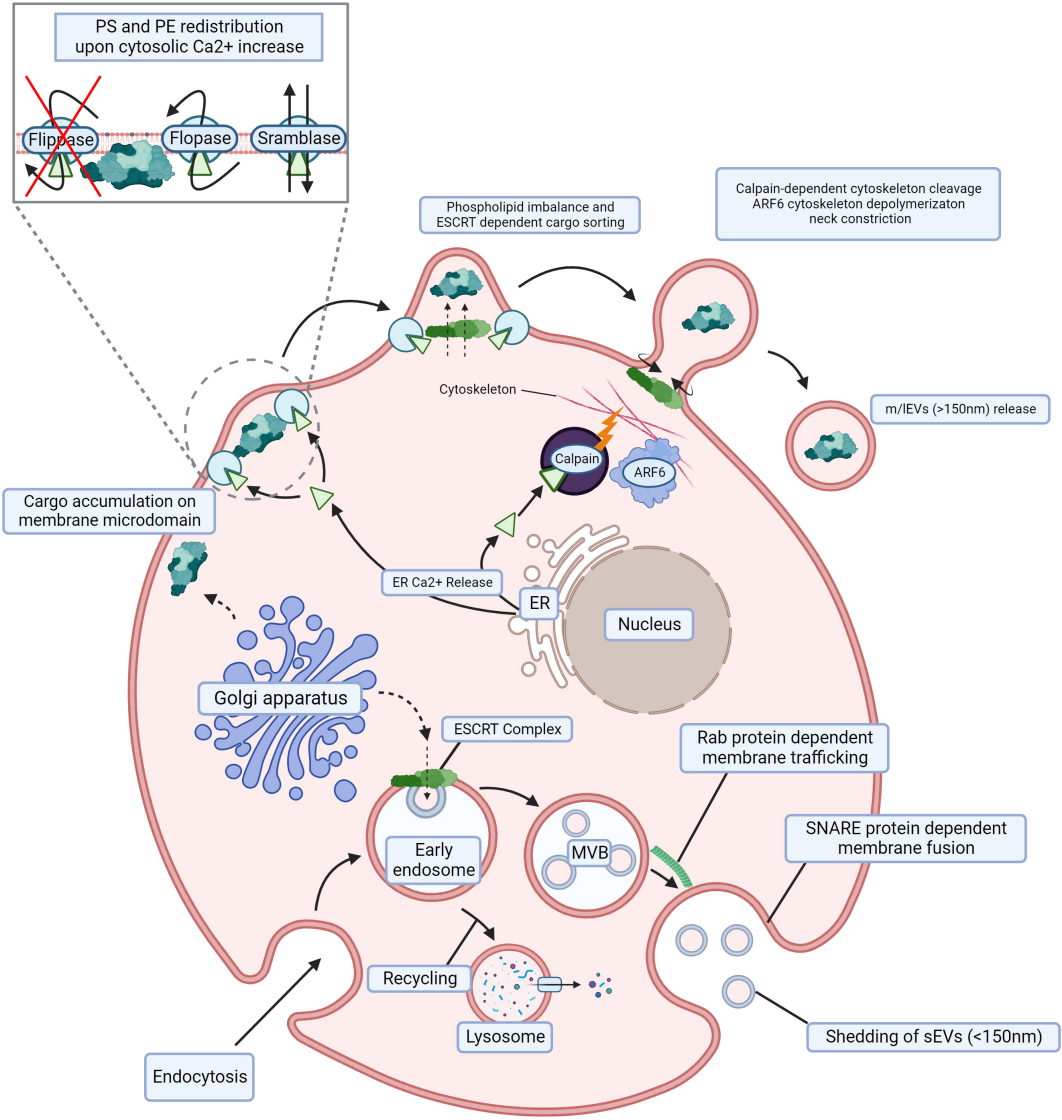
Biogenesis of m/lEVs and sEVs. m/lEVs arise from membrane budding independent of the endocytic compartment, whereby phospholipid and cytoskeletal disruption are the main drivers of their shedding. Cargo aggregates below the cell surface, and Ca^2+^ release from the ER activates floppase, scramblase and calpain while simultaneously deactivating flippase. This redistributes PS and PE on the cell membrane and leads to the disruption of membrane cytoskeletal interactions. The absence of such interactions facilitates spontaneous membrane budding, resulting in m/lEV release. sEVs originating from the endocytic compartment. Following endocytosis, ILVs are generated and subsequently released through trafficking and fusion of the late endosome also called multivesicular bodies (MVB) with the cell membrane. An early endosome is internalized through endocytosis by the cell, and its content can thereafter be recycled in a lysosome. The ESCRT complex and accumulation of sphingolipid ceramide allow for the formation of ILVs within the MVB. Subsequent Rab protein-dependent trafficking toward the cell membrane and SNARE protein-dependent fusion with the cell membrane are required for the shedding of ILVs (sEVs). m/lEVs, medium to large extracellular vesicles; sEVs, small extracellular vesicles; ER, endoplasmic reticulum; PS, phosphatidylserine; PE, phosphatidylethanolamine; ILV, intraluminal vesicle; MVB, multivesicular body; ESCRT, endosomal sorting complex required for transport; SNARE, snap receptor. Created with BioRender.com.

**Figure 2:**
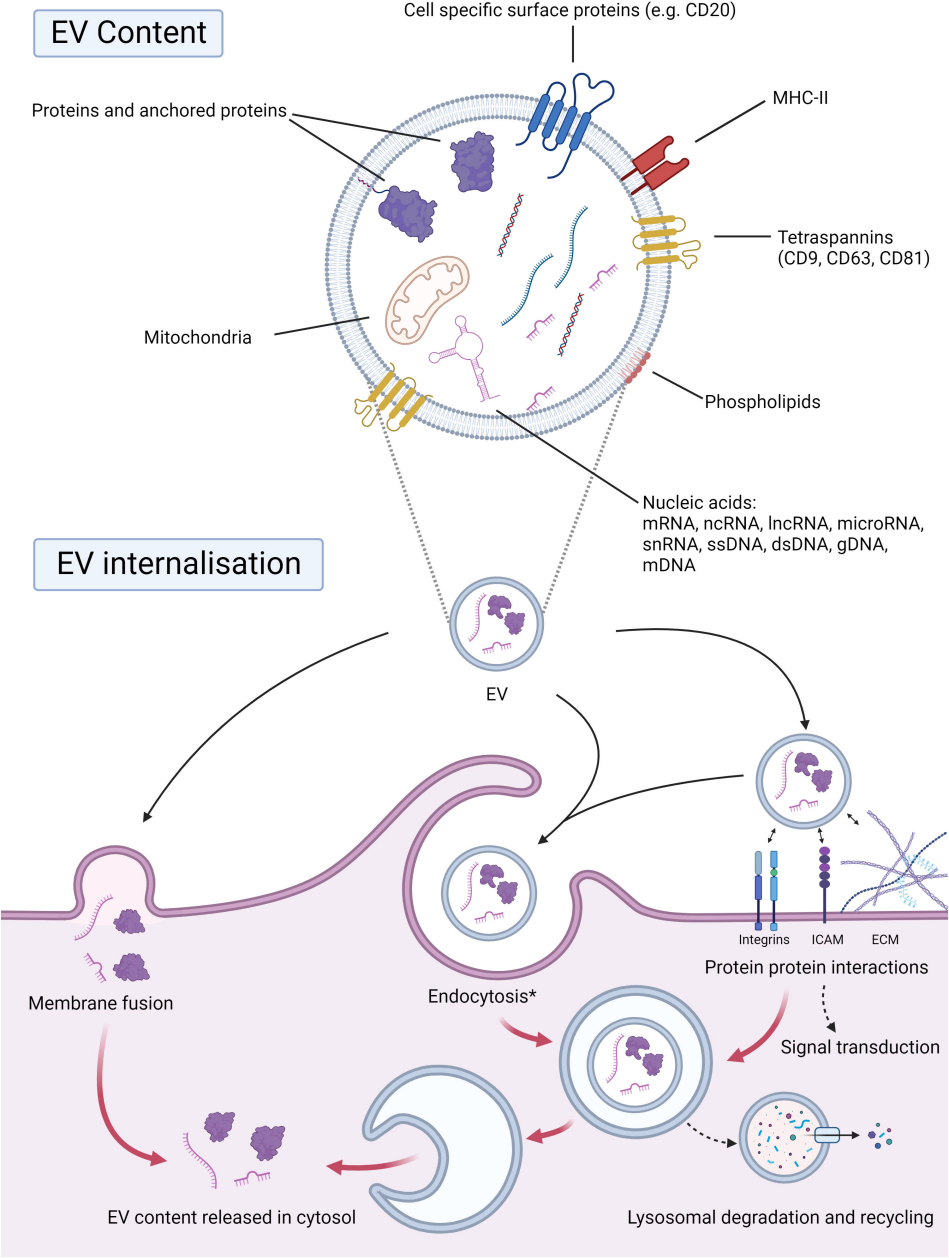
EV cargo and internalization. EVs encapsulate selected cargo within their phospholipid bilayer, which is often representative of shedding cells. Proteins within the EV, anchored or even surface proteins, are integral for the downstream signaling and internalization of EVs. Surface proteins such as tetraspanins (CD9, CD63, CD81), MHC-II, or even cell-specific proteins, e.g., CD20, may be present. The phospholipid composition of EVs will also be transferred upon internalization by receptive cells. Nucleic acids represent a large portion of the cargo transported by EVs. EVs may carry nucleic acid species such as mRNAs, ncRNAs, lncRNAs, microRNAs, ssDNAs, gDNAs, mDNAs, and snRNAs. Mitochondria and ATP can also be transported by EVs and brought to the receptive cell, altering its energy metabolism. EVs can be internalized by direct membrane fusion, whereby their cargo will be transferred to the cytosol. EVs may also interact with surface proteins and the receptive cell’s extracellular matrix, which can lead to downstream signal transduction and/or endocytosis. The contents may thereafter be recycled through lysosomal degradation or released in the cytosol. *Endocytosis: micropinocytosis, phagocytosis, classical endocytosis. Abbreviations: EV; extracellular vesicle, CD; cluster of differentiation, MHC-II; major histocompatibility complex class II, mRNA; messenger RNA, ncRNA; non-coding RNA, lncRNA; long non-coding RNA, ssDNA; single stranded DNA, gDNA; genomic DNA, mDNA; mitochondrial DNA, snRNA; small nuclear RNA. Created with BioRender.com.

Although the cell of origin plays an immense role in EV cargo composition, the selection of molecules to be packaged is a highly regulated process. Specific sorting machinery ensures that proteins, nucleic acids (DNA, RNA, non-coding RNA) and glycolipids carried by EVs drastically differ from the cell of origin (9). Selective protein packaging is dependent on posttranslational modifications, particularly ubiquitination and sumoylation, that target specific proteins to be sorted by the endosomal sorting complex required for transport (ESCRT) machinery (10, 11). In addition to the presence of major histocompatibility complex-II (MHC-II) molecules on the EV surface, tetraspanins are enriched on EVs compared to the lysosomal membrane and plasma membrane (5, 12). Notably, CD63, CD81 and CD9 have been observed to be enriched on sEVs originating from diverse cell types (13). However, the presence of these tetraspanins has been detected on m/lEVs as well (14). In a proteomic comparative study, Kowal et al. demonstrated, on one hand, that sEVs are also enriched in Annexin XI (but also Annexins V and VI), ACE (angiotensin-converting enzyme), syntenin-1 [involved in receptor targeting to intraluminal vesicles of multivesicular bodies (15)], TSG101 (Tumor Susceptibility 101, a ESCRT pathway protein), ADAM10 (ADAM Metallopeptidase Domain 10), and EHD4 (EH Domain Containing 4), syntenin-1 and TSG101 being specific of the tetraspanin-enriched sEVs representing exosomes (16). On the other hand, GP96 (Glycoprotein 96) and possibly other ER-associated proteins are mainly present in large EVs while actinin-4 and mitofilin, and possibly other mitochondrial proteins, are present in both large and medium-sized EVs but absent from the sEVs. In addition, proteins associated with the outward budding cause notable differences in the proteomic profile of m/lEVs compared to their sEV counterpart. For instance, m/lEVs containing gelatinases, ARF6, MHC-1, MT1MMP, VAMP3 and β1-integrin likely originate from a myosin light-chain kinase (MLCK) cascade dependent release (17). Alternatively, other pathways involved in m/lEV release such as RAB22A dependent secretion and ARRDC1/TSG101/VSP4 dependent shedding result in TGM2 containing (18) and TSG101/ARRDC1 containing m/lEVs respectively (19). Outside of proteins associated with the biogenesis of EVs, a 2021 report exploring available EV datasets describes the tendency of m/lEVs to carry conserved proteins across prokaryotes and eukaryotes and related to mitochondrial function or energy synthesis, in symphony with the conserved process of membrane budding (20). sEVs on the other hand contain proteins more akin to the recent emergence of the endosomal vesiculation process. These proteins were part of signaling pathways or involved in the synthesis of intracellular building blocks (20). sEVs and m/lEVs also carry various RNA species: messenger RNA (mRNA) (21), non-coding RNAs (ncRNAs) such as microRNAs (miRs) along with their precursors (pre-miRNAs) (22), Y-RNAs (23, 24), small nuclear RNAs (snRNAs) (25), long non-coding RNAs (25, 26), piwi-interacting RNAs (21, 25), small nucleolar RNAs (21, 25), ribosomal RNAs (21, 25), transfer RNAs (21, 25), mitochondrial RNAs (27), circular RNAs (circRNAs) (28, 29) and vault RNAs (30). Among the RNAs encapsulated within human plasma derived EVs, microRNAs are the most abundant and have been described to play a significant role in cancer by promoting tumor progression and ME immunosuppression (25, 31). DNA species such as single-stranded DNA (ssDNA), double-stranded DNA (dsDNA), genomic DNA (gDNA) and mitochondrial DNA (mDNA) have been observed in sEVs; however, whether DNA packaging into sEVs is selective remains unclear (32). An overview of EV biogenesis, cargo and the process of internalization are illustrated in **Figures 1**, **2**.

## Hematological malignancies

A total of 186,400 people are estimated to have been diagnosed with leukemia, lymphoma, or myeloma in the US in 2021; combined, these new cases represent 9.8% of cancer cases diagnosed in 2021 (33). Here we will review the role of EVs in different diseases: acute myeloblastic leukemias (AML), myeloproliferative neoplasms, acute lymphoblastic leukemias (ALL) and lymphoproliferative neoplasms.

## Myeloid neoplasms

### Acute myeloid leukemia

Characterized by the uncontrolled proliferation of myeloblasts or progranulocytes that do not differentiate properly, acute myeloid leukemia (AML) is an aggressive heterogeneous hematological malignancy (34). Diagnosis is established through the examination of a peripheral blood sample or bone marrow biopsy where the presence of circulating blasts is assessed. The presence of excess immature myeloid cells (>20% blasts) in the sample by flow cytometry confirm the diagnosis (34, 35).

**Figure 3:**
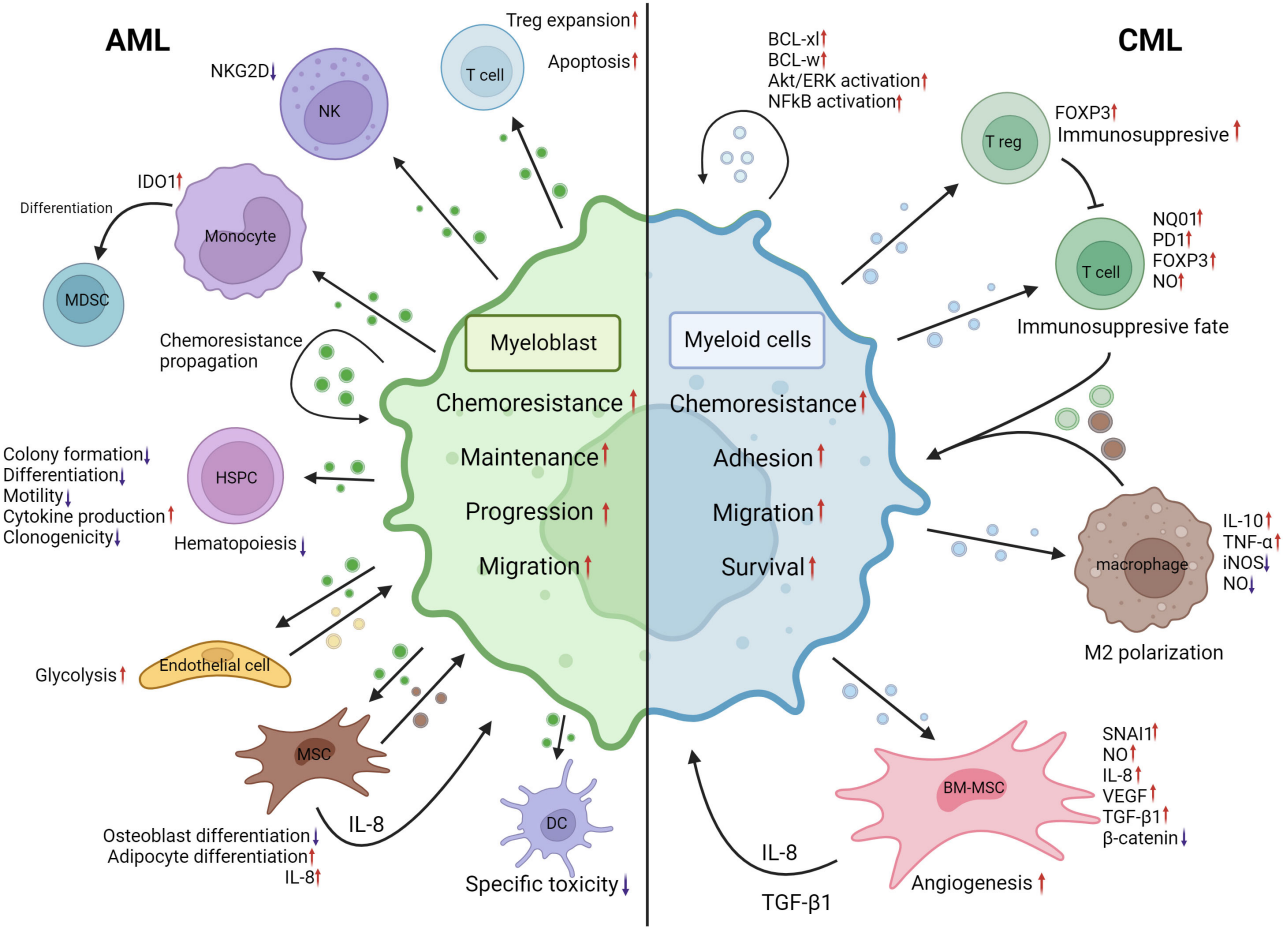
EVs mediated crosstalk between AML myeloblasts, CML myeloid malignant cells and their MEs. AML myeloblast EVs induce Treg expansion and apoptosis in the T-cell population. NKG2D expression is decreased in NK cells following AML EV internalization. Monocyte IDO1 expression is increased and differentiation into MDSCs is induced by AML EVs. In an autocrine manner, malignant AML cells may propagate chemoresistance by means of EVs. HSPC colony formation, differentiation, motility and clonogenicity are negatively impacted by AML EVs, resulting in hematopoiesis disruption. However, inflammatory cytokine production is increased. AML EVs induce increased glycolysis in endothelial cells resulting in heightened vascular remodeling. BM-MSCs internalizing AML EVs induced adipocyte differentiation and IL-8 production; however, osteoblast differentiation was negatively impacted. The specific toxicity of dendritic cells is decreased following interactions with AML EVs. Increased IL-8 production by BM-MSCs, BM-MSC EVs, endothelial cell EVs and ME changes induced by AML EVs contribute to increased chemoresistance, maintenance, progression and migration of myeloblasts. CML cell EVs can induce BCL-xl, BCL-w, AKT/ERK and NF-kB activation in an autocrine manner. Upon internalization, CML cell EVs induce NQ01, FOXP3 and PD1 expression increases whilst increasing NO production in T cells, leading to an immunosuppressive fate. CML EVs facilitate M2 polarization of macrophages by inducing IL-10, TNF-α expression and decrease iNOS expression and NO levels. CML EVs, when internalized by BM-MSCs, can cause a decrease of β-catenin and increased NO levels, IL-8, SNAI1 and VEGF expression. These changes promote angiogenesis, IL-8 secretion and TGF-β1 secretion. Changes brought by CML EVs to ME cells lead to increased chemoresistance, adhesion, migration, and survival of malignant cells. CML, chronic myelogenous leukemia; AML, acute myeloid leukemia; BCL-xl, B-cell lymphoma extra large; BCL-w, BCL2 like2; AKT, Serine/Threonine kinase; ERK, mitogen activated protein kinase 1; NF-kB, Nuclear factor kappa B; NQ01, NADPH dehydrogenase 1; FOXP3, forkhead box P3; PD1, programmed cell death 1; NO, nitric oxide; IL-10, interleukin-10; TNF-α, tumor necrosis factor alpha; iNOS, nitric oxide synthase; BM-MSC, Bone marrow mesenchymal stromal cell; IL-8, interleukin-8; SNAI1, snail family transcriptional repressor 1; VEGF, Vascular endothelial growth factor; ME, Microenvironment; NKG2D, Killer Cell Lectin Like Receptor K1; IDO1, Indoleamine-2,3-Dioxygenase 1; MDSC, Myeloid derived suppressor cell; HSPC, hematopoietic stem and progenitor cell. Created with BioRender.com.

The creation and maintenance of leukemic niches is crucial for immature myeloid blast relocation to secondary bone marrow sites. Circulating leukemic cells can invade tissues and pioneer new bone marrow niches by modulating the ME. AML cells secrete sEVs that can reshape the immune response, increase chemoresistance facilitating disease progression (36, 37). When treated with tumor-derived EVs, Wieckowski et al. observed a decrease of signaling protein such as JAK3 and CD3ζ in activated T cells as well as decreased phosphorylation of STAT5 in activated CD8+ cells while a decrease is observed in activated CD4+ cells (38). In addition, these authors observed an enrichment of FASL (Fas cell surface receptor ligand) and MHC class I molecules in tumor – derived m/lEVs. Interestingly, tumor cell line derived-EVs significantly affected CD8+ T cell expansion and improved Treg proliferation. Hong et al. also shows that these EVs are able to induce apoptosis in CD8+ T cells (39, 40). Further, tumor-derived sEVs inhibit CD8+ T cell SLC6A8 (solute carrier family 6 member 8) dependent creatine import in NPM-1 mutated AML cells through the transfer of miR-19a-3p (41). Indeed, as a target of miR-19b-3p, SLC6A8 expression is diminished following sEV internalization which hampers creatine import, thereby affecting CD8+ Tcell immune function (41). In addition to the effect on effector T cells, NKG2D expression in NK cells was decreased following the addition of AML patient sera derived EVs (40), thereby reducing their potential activity. This effect was thwarted upon the addition of neutralizing anti TGF-β1 antibodies or when supplementing the NK cells with IL-15 (42). Besides their effect on T and NK cells, AML-derived EVs also stimulate monocytes through the presence of palmitoylated proteins on the EV surface to undergo differentiation into myeloid-derived suppressor cells (MDSC) (43). Following coculture with AML-derived EVs, Tohumeken et al. reported increased suppressive activity and indoleamine-2,3-dioxygenase (IDO1) gene upregulation in HL60-primed monocytes (43). Dendritic cells (DCs) treated with AML patient-derived EVs in comparison to K562 (chronic myelogenous leukemia cell line)-derived EVs were shown to have a notable decrease in specific cytotoxicity, further contributing to the immunosuppressive environment fostered by the malignancy (44). In addition to immune response modulation, AML-derived sEVs can shape the bone marrow niche into a leukemia-permissive ME, hampering normal hematopoiesis (45, 46). AML-derived sEVs were shown to have increased dipeptidyl-peptidase 4 (DPP4) activity compared to healthy donors. DPP4, implicated in hematopoiesis regulation, was enriched in AML-derived sEVs and severely inhibited hematopoietic progenitor cell (HPC) colony formation. Furthermore, HPC differentiation was significantly restricted upon treatment with AML-derived sEVs (47). In accordance with these findings, a transcriptomic study of HSPCs following treatment with AML-derived sEVs found 923 downregulated genes and 630 upregulated genes involved in hematopoiesis and differentiation (45). These sEVs were also observed to impact the clonogenicity, inflammatory cytokine production, C-X-C chemokine receptor type 4 (CXCR4) expression, and Stromal cell-derived factor 1 (SDF1)-mediated migration of hematopoietic stem and progenitor cells (HSPCs). These changes were accompanied by an accumulation of less differentiated myeloid progenitors (45). Additionally, the enrichment of miR-150 (48), miR-155 (48), miR-584ac (46) and miR-4532 (49) in AML sEVs was linked to the suppression of MYB proto-oncogene (c-MYB), tripartite motif containing 28 (TRIM28) and leucine zipper downregulated in cancer (LDOC1) expression respectively, modulating HSPC hematopoiesis (46, 48, 49). Furthermore, human-to-mouse AML-derived sEVs were observed to significantly reduce osteoblast differentiation while increasing adipocyte differentiation in AML mouse models supporting by this way the leukemic stem cell metabolic needs, strengthening the leukemic niche and promoting disease progression (50). The switch in differentiation brought by AML-derived EVs in BM-MSCs promotes increased marrow fat content that linked to enhanced chemoresistance (50). VEGF and VEGF mRNA cargo in these vesicles also induce glycolysis in human umbilical vein endothelial cells (HUVECs) and promote vascular remodeling, further increasing AML chemoresistance (51). Endothelial cells themselves, with the release of angiopoietin-like 2 (ANGPTL2)-containing EVs, are able to accelerate AML progression through the interaction between ANGPTL2 and leukocyte immunoglobulin-like receptor B2 (52). Moreover, BM-MSC-derived EVs in AML have been shown to participate in AML disease progression and increase chemoresistance (53–56). In fact, following the uptake of BM-MSC-derived EVs, S100 calcium binding protein A4 (S100A4) expression is increased, and glycogen synthase kinase-3 beta (GSK-3β) expression is decreased through miR-26a-5p EV enrichment, leading to increased proliferative capability, migration and chemoresistance (53, 54). Alternatively, fibroblast growth factor 2 (FGF2) packaged within BM-MSC EVs protects AML leukemic cells against tyrosine kinase inhibitors (TKIs) through heightened FGF2-FGFR signaling, which participates in stromal growth and paracrine protection (55). Additionally, KG1A (an AML cell line)-derived EVs could induce increased IL-8 production in BM-MSCs, which was linked to the diminished chemosensitivity of the AML cells (56). On the other hand, several studies have described an anti-leukemic effect of MSC-derived EVs by transferring miR-124-5p (57), miR-222-3p (58), miR-7-5p (59), miR-146a-5p (60) and miR-23b-5p (61) to AML leukemic cells resulting in enhanced apoptosis, cell cycle arrest, proliferation suppression and increased chemosensitivity (57–61). Conversely, circular RNAs circ_0009910 (28) and circ_0004136 (29) are both enriched in AML-derived EVs, promote AML malignant cell progression, reduce apoptosis and promote proliferation by acting as sponges for miRs targeting growth factor receptor bound protein 10 (GRB10) and tetraspanin 3 (TSPAN3), respectively (28, 29). Furthermore, miR profiling of AML-derived EVs revealed a distinct prolife, with deregulated miRs able to target known AML-related factors such as GSK-3β and Enhancer Of Zeste 2 Polycomb Repressive Complex 2 Subunit (EZH2) (62). Recently, Hong and colleagues described that AML-derived sEVs promote chemoresistance by an autocrine mechanism: AML blasts become chemoresistant due to the presence of 3-hydroxy-3-methyl-glutaryl coenzyme A reductase (HMGCR) on AML-derived sEVs, which upregulates cholesterol synthesis followed by a massive release of HMGCR+ sEVs into the extracellular space, perpetuating this vicious cycle that acts against the efficacy of chemotherapy (63). Similarly, apoptosis-resistant AML blasts can induce *de novo* chemoresistance in naïve blasts through the transfer of EVs by altering gene regulatory networks or inducing drug efflux pump expression and reducing reactive oxygen species (ROS) generation in target cells (64). AML BM-MSC EVs also contribute to the chemoresistance of AML cells through the delivery of miR-10a, which targets regulation of nuclear pre-mRNA domain containing 1A and the Wnt/β-catenin pathway. Downregulation of miR-10a significantly increased the sensitivity of AML cells to cytarabine treatment (65). Resistance to decitabine, a commonplace front-line therapy for AML patients, is promoted by the transfer of miR-4755-5p within EVs from KG1a decitabine resistant cells to other KG1a cells (AML cell line). Following this transfer, CDKN2B, a cyclin dependent kinase inhibitor displays diminished expression indicating direct targeting by miR-4755-5p (66). Further highlighting the importance of sEV shedding in the AML ME, miR-34c-5p, a microRNA downregulated in AML, was described to induce leukemic stem cell (LSC) senescence and promote the eradication of LSCs (67). MiR-34c-5p could also be part of a positive feedback loop along with Rab27B, perpetuating the increase in its own intracellular level by blocking sEV-mediated export (67). Conversely, miR-1246, enriched in AML cell-derived EVs, through its targeted repression of leucine rich repeats and immunoglobulin-like domains 1 (LRIG1), activates the signal transducer and activator of transcription 3 (STAT3) pathway, thereby increasing the viability and differentiation of LSCs (68). Previously mentioned reports have been summarized in **Figure 3**.

### Myeloproliferative neoplasms

#### Chronic myeloid leukemia

Characterized by the unimpeded growth of myeloid cells at different maturation stages, chronic myeloid leukemia (CML) is a myeloproliferative neoplasm mainly occurring in the older population, with a median age of 57 years old (69).

In chronic myeloid leukemia (CML), it is currently well known that signals originating from the microenvironment can promote cell proliferation, survival and chemoresistance. Indeed, CML cells can release EVs that promote malignant cell expansion in a dose-dependent autocrine manner *in vitro* and *in vivo* (70). The expression of antiapoptotic genes such as survivin, B-cell lymphoma extra-large (BCL-xl) and B-cell lymphoma w (BCL-w) and activation of serine/threonine kinase/extracellular regulated kinase (Akt/ERK) and NFκB pathways is significantly increased in CML cells treated with CML-derived sEVs (70). Other cell types may take up EVs originating from leukemic cells in CML. The uptake of CML-derived sEVs by immune cells and stromal cells in the ME can in turn influence the ME to diverge toward a leukemic niche. Through modulation of cytokine release and redox potential of BM-MSCs and macrophages, CML-derived EVs alter the leukemic niche by pushing it towards an immunosuppressive environment, promoting disease progression (71, 72). Following exposure to K562 (CML cell line)-derived EVs, T cells demonstrate a phenotypic shift toward an immunosuppressive fate, upregulating signal transducer and activator of transcription 5 (STAT5) signaling whilst decreasing mammalian target of rapamycin – ribosomal s6 kinase (mTOR-S6) signaling leading to a marked Forkhead box P3 (FoxP3) upregulation (73). Other genes associated with immunosuppressive phenotypes such as NAD(P)H dehydrogenase Quinone 1 (NQ01) and Programmed cell Death protein 1 (PD1) were also shown to be induced in cord blood derived T-cells when treated with K562- derived EVs (71). The authors noted a simultaneous decrease in CD3 Delta Subunit Of T-Cell Receptor Complex (CD3d) and Nuclear Factor of Activated T Cells 3 (NFATc3) expression, associated with T cell activation, and significant changes in cytokine production and intracellular ROS levels (71). Mouse macrophages, internalizing CML-EVs, display expression changes in genes associated with macrophage polarization such as tumor necrosis factor-α (TNF-α) and interleukin-10 (IL-10) that are markedly upregulated, while nitric oxide synthase (iNOS) expression decreased, in line with M2 tumor-associated macrophage characteristics (72). BCR-ABL1 mRNA cargo in K562- EVs can induce increased expression of BCR-ABL1 and TGF-β1 in BM-MSCs indirectly promoting K562 proliferation (74). Likewise, amphiregulin (AREG) presence on the LAMA84 cell line-derived EV surface could induce epidermal growth factor (EGFR) phosphorylation on BM-MSCs, resulting in increased Snail family transcriptional repressor 1 (SNAI1) expression. Interleukin-8 (IL-8) and matrix metalloproteinase 9 (MMP9) are known targets of SNAI1 and were shown to exhibit increased expression following EV treatment of BM-MSCs and a stromal cell line (75). Importantly, IL-8 released by BM-MSCs can promote the adhesion and migration of malignant cells, enhancing their survival (76). Wong and colleagues recently demonstrated that K562-derived sEVs carrying the long non-coding RNA LNC000093 and the microRNA miR-675-5p regulate vascular endothelial growth factor (VEGF) expression in BM-MSCs (77), coinciding with the previously described angiogenic ability and high level of VEGF in CML patient plasma that contribute to the tumorigenic niche (78). The growth-suppressive miR-320 is selectively sorted in CML EVs by RNA binding protein heterogeneous nuclear ribonucleoprotein A1 (hnRNPA1), transferred to BM-MSCs and inhibits β-catenin, thereby hindering osteogenesis, remodeling of the BM niche and promoting CML progression (79). Additionally, the selective sorting of miR-320 into sEVs results in a alleviating BCR-ABL repression, and allowing for increased proliferation of donor CML cells (79). A 2021 study demonstrated enrichment of the circular RNA (circRNA) hsa_circ_0058493 in CML patient peripheral blood mononuclear cells (PBMCs) and the sEVs they secrete. Increased hsa_circ_0058493 expression coincides with the rise in CML cells resistant to the tyrosine kinase inhibitor imatinib (80). Moreover, Zhang and colleagues studied the effect of BM-MSC-derived sEVs on CML cell chemoresistance (81). CML cells quickly demonstrated diminished viability following treatment with imatinib. Conversely, viability was restored when CML cells were incubated with both BM-MSC-derived sEVs and imatinib, indicating that EVs originating from the ME can induce chemoresistance in CML cells (81). In an autocrine manner, CML EVs originating from imatinib-resistant cells can confer imatinib resistance to imatinib-sensitive CML malignant cells upon transfer (82). The implications of EVs in CML have been summarized in **Figure 3**.

### Primary myelofibrosis

Primary myelofibrosis (PMF) is a rare myeloproliferative neoplasms (MPN) characterized by mutations in three driver genes that cause an hematopoietic stem cell – derived monoclonal proliferation: janus kinase 2 (JAK2), calreticulin (CALR) or MPL proto-oncogene (MPL) (83). Abnormal hematopoiesis and the formation of fibrous tissue within the bone marrow are hallmarks of PMF and is linked to altered cytokine profile in the bone marrow (84, 85).

Caivano et al. showed that the number of EVs lower than 0.3 μm are significantly higher in PMF patient than in healthy controls (86). Ruxolitinib, a JAK2 inhibitor frequently used for MPN patient treatment was shown to shift the proportionate number of m/lEVs shed by platelets towards a greater part being released by megakaryocytes (87). Barone et al. highlight the decreased ability of JAK2V617F-mutated PMF patient monocytes to secrete EV-linked cytokines (such as IL-1β, TNF-α, IL-6, IL-10) under lipopolysaccharides (LPS) stimulation. Interestingly, this decrease of cytokines was not observed in ruxolitinib-treated patients and the ability of PMF monocytes was restored after *in vitro* ruxolitinib treatment (88). In addition, EVs from PMF patients carry mitochondrial components (highlighted by the MitoTracker dyes) suggesting that these EVs could potentially fuel tumor cell maintenance and proliferation (89). However, additional investigation to understand these mitochondrial components are needed.

## Lymphoid neoplasms

### Precursor B-cell neoplasm

Acute lymphoid leukemia (ALL) is a rare type of leukemia generally diagnosed under 20 years old and mainly impacting children under 4 years old (90). ALL is characterized by the uncontrolled accumulation of immature (i.e., largely undifferentiated) B- or T-lymphocyte precursors that can invade the bone marrow, blood, and extramedullary sites (90). B-lymphocyte ALL (B-ALL) is the most common of the two subtypes, with T-ALL representing only 12%-15% of newly diagnosed cases in pediatric patients (91). Approximately 25% of B-ALL cases are subtyped as Philadelphia chromosome-positive ALL cases. Due to a translocation of chromosome 9 and 22 t(9,22) the genes BCR and ABL1 fuse, forming BCR-ABL1, a fusion tyrosine kinase facilitating cell division and disease progression (92). The translocation t(9,22) is a leading factor for a poor prognosis of B-ALL patients associated with higher white blood cell counts and older age features (93).

**Figure 4:**
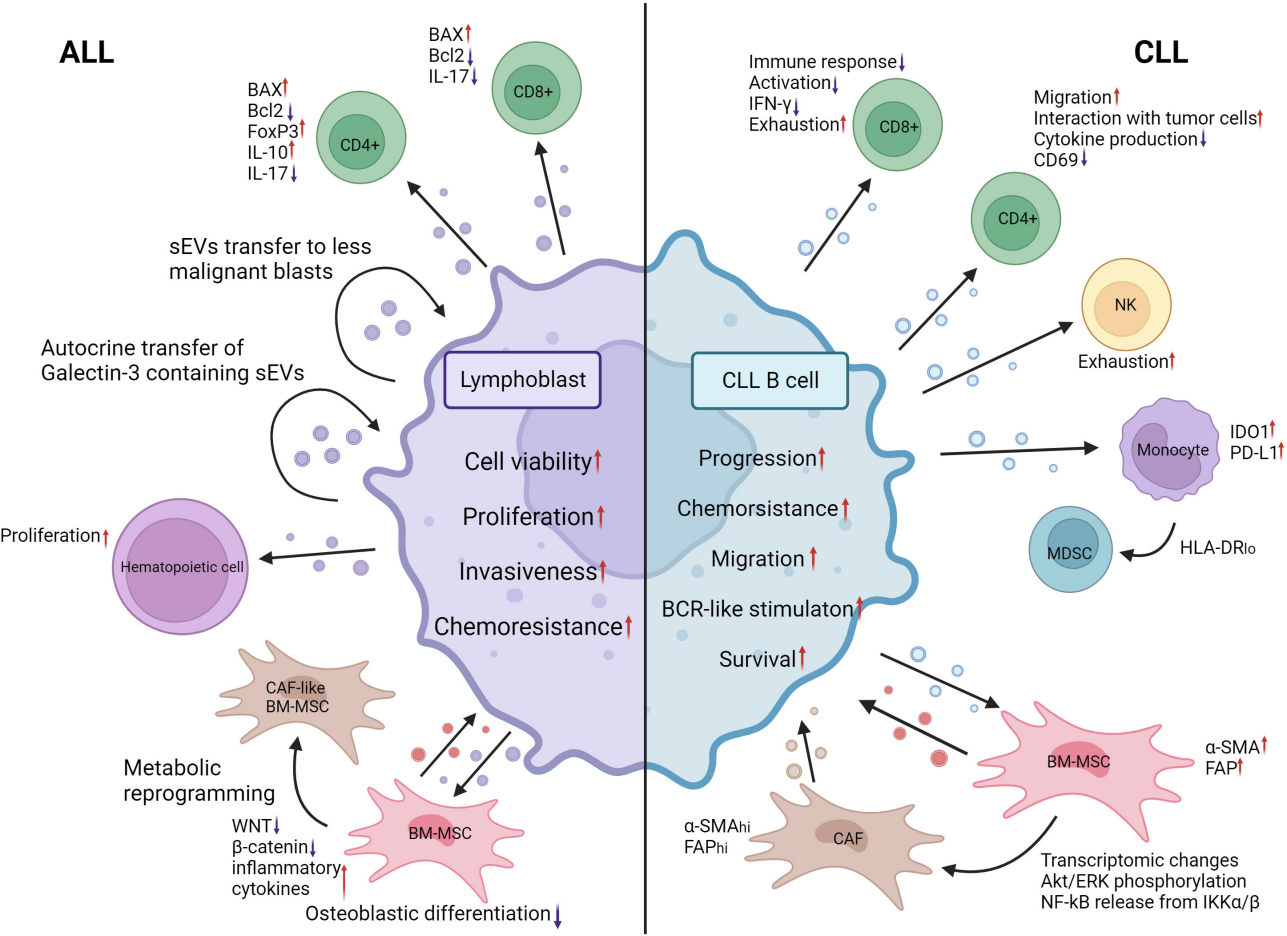
EV mediated crosstalk between ALL lymphoblasts, CLL malignant B cells and their MEs. ALL lymphoblasts shed EVs that upon internalization by CD8^+^ and CD4^+^ Th17 T cells promote apoptosis by inducing BAX expression and decreasing Bcl2 expression. Likewise, a significant uptick of FoxP3 expression and immunosuppressive cytokine secretion was observed T cells after 24h of coincubation with ALL EVs. ALL EVs containing galectin-3 are transferred to other ALL blasts in an autocrine manner, promoting disease progression and chemoresistance. Hematopoietic stem cell proliferation is increased following treatment with ALL lymphoblast EVs. WNT and β-catenin expression in BM-MSCs is decreased following ALL EV internalization. The production of inflammatory cytokines is increased. Their osteoblastic differentiation is significantly decreased. Metabolic reprogramming of BM-MSCs toward a CAF-like phenotype was also observed. The autocrine signaling and BM-MSC reprogramming induced by AML EVs promote the viability, proliferation, invasiveness and chemoresistance of ALL blasts. CLL malignant B-cell EVs induce exhaustion of CD8 T cells by reducing their activation and production IFN-γ. CD4 T cells also undergo decreased production of cytokines and expression of CD69 when treated with CLL EVs. Their migratory capacity and interaction with malignant cells are increased. BAG6 rich CLL EVs induce exhaustion in NK cells, reducing their activity. IDO1 and PD-L1 expression in monocytes is increased following internalization of CLL EVs. CLL EVs also contribute to the expansion of the HLA-DR-low MDSC population. BM-MSCs undergo increased expression of α-SMA and FAP when exposed to CLL EVs. Combined with other transcriptomic changes and activation of signaling pathways, they transform into cancer-associated fibroblasts that are essential for CLL expansion through the release of EVs. Modification of the ME by CLL EVs contributes to the progression of malignancy, chemoresistance, migration, survival and BCR stimulation. CLL, Chronic lymphocytic leukemia; ALL, Acute lymphocytic leukemia; IFN-γ, Interferon gamma; CD69, cluster of differentiation 69; BAG6, BAG chaperone 6; IDO1, Indoleamine-2,3-Dioxygenase 1; PD-L1, Programmed death ligand 1; HLA-DR, HLA Class II Histocompatibility Antigen DR; MDSC, Myeloid derived suppressor cell; α-SMA, Alpha smooth muscle actin; FAP, Fibroblast activation protein alpha; AKT, Serine/Threonine kinase; ERK, mitogen activated protein kinase 1; NF-kB, Nuclear factor kappa B; IKKα/β, inhibitor of nuclear factor kappa-B kinase subunit beta; CAF, cancer associated fibroblasts; BCR, B-cell receptor; WNT, WNT family member 1. Created with BioRender.com.

Interactions between Acute lymphoid leukemia (ALL) blasts and the bone marrow ME are crucial to maintain malignant cell self-renewal, expansion, and chemoresistance necessary for leukemic development (78, 94–98). Pleckstrin homology domain family M member 1 (PLEKHM1) deficiency in the bone marrow microenvironment accelerated BCR-ABL^+^ B-ALL disease progression by altering MSC EV cargo (98). TNF-α secretion by B-ALL cells cause PLEKHM1 deficiency in MSCs that release sEVs increasing the number and improving the function of leukemia initiating cells through the transfer of enriched synthenin and syndecan-1 (98). As a result of this transfer, phosphorylation of AKT and focal adhesion kinase occurs, which in turn promotes differentiation, migration, and disease progression in BCR-ABL^+^ B- ALL (98). The osteogenic differentiation of BM-MSCs is inhibited by miR-34a-5p carried by sEVs released by T-ALL blasts. Yuan and colleagues described overexpression of miR-34a-5p in T-ALL patient cells, in cell lines and in their corresponding sEVs (99). Furthermore, miR-34a-5p overexpression coincides with reductions in WNT and β-catenin expression and, consequently, osteogenic differentiation (99). Hematopoietic stem cell populations are gravely affected by ALL shed EVs, altered cytokine secretion, exhaustion, and failure to maintain HSC niche can be caused by these vesicles (94, 97, 100). Overexpressed miRs in ALL such as miR-146a-5p, miR-181b-5p and miR-199b-3p were shown to functionally bind to TLR8 in hematopoietic cells and BM-MSCs, promoting NF-kB activation and the subsequent secretion of proinflammatory cytokines aiding in the expansion of early hematopoietic cell populations (94). Eventually, this interaction leads to the establishment of a tumor microenvironment-hematopoietic feedback loop facilitating disease progression (94). Furthermore, Heat shock protein 70 (HSP70)^+^ ALL blast EVs rich in cholesterol target murine HSPCs in an *ex vivo* model and disrupt their quiescence by altering their mitochondrial energy metabolism and leading to an eventual exhaustion of these cells thereby affecting hematopoiesis (97). Additionally, through protein kinase RNA-like endoplasmic reticulum kinase (PERK) dependent Jagged canonical Notch ligand 1 (JAG1) upregulation brought by T-ALL EVs, SCF and CXCL12 expression is markedly suppressed jeopardizing HSC maintenance and regeneration (100). After 24h of exposure, ALL patient serum derived EVs may also induce an immunosuppressive environment by hindering T cell immunity. Upon internalization by CD8^+^ and CD4^+^ Th17 T cells, ALL EVs promote apoptosis by inducing BAX expression and decreasing Bcl2 expression (101). Likewise, a significant uptick of FoxP3 expression and immunosuppressive cytokine secretion was observed in T cells after 24h of coincubation with ALL EVs (101). In ALL patient samples, miR-181b-5p was significantly overexpressed in ALL blast-derived EVs compared to healthy donors and this overexpression may act in an autocrine manner to promote the proliferation and migration of other ALL cells (102). ALL3 cells (a Philadelphia chromosome positive ALL cell line) do not grow in low density conditions, however sEVs released by ALL3 cells alone could mimic a high-density environment, restoring these conditions and improving the proliferation and viability of the cell line (95). ALL cells co-cultured with stromal cells were described to contain high relative galectin-3 levels. Fei et al. postulate that internalization of stromal-derived sEVs containing galectin-3 by ALL cells may be a major contributor to ALL cell galectin-3 content. Increased galectin-3 content support stressed B-ALL cells during chemotherapy by inducing tonic NF-κB pathway activation (103). Proteomic profiling of plasma sEVs in B-ALL reveal 342 differentially expressed proteins associated with metabolic proceses and protein activity regulation (104). Further, the differentially expressed proteins were associated with the NOTCH receptor 1 (NOTCH1) and autophagy pathways (104). Furthermore, miRs downstream of NOTCH receptor 1 (NOTCH1) signaling in T-ALL have been identified as mostly comprised of members of the mir-17-92a cluster and their paralogues (96). EVs secreted by T-ALL cells are enriched with these miRs, which can counteract γ-secretase inhibitor dependent blocking of T-ALL proliferation *in vitro*, underlining the importance of EV mediated chemoresistance in ALL (96). Previously mentioned reports have been summarized in **Figure 4**.

### Mature B-cell neoplasms

#### Chronic lymphocytic leukemia

Marked by the accumulation of small mature CD5+/CD19+/CD23+ B-lymphocytes in the peripheral blood, bone marrow, spleen and lymph nodes, chronic lymphocytic leukemia (CLL) is the most diagnosed leukemia in adults of Western countries (105, 106). CLL diagnosis requires the detection of ≥5,000 B-lymphocytes/µL of peripheral blood for a minimum of 3 consecutive months (105). Rai et al., Binet et al. and CLL-international prognostic index (CLL-IPI) classifications stratify the patient into risk categories ranging from low risk with little to no treatment to high-risk patients requiring immediate treatment (107–109).

The interaction between chronic lymphocytic leukemia (CLL) B-lymphocytes and the ME is essential in disease maintenance and progression. CLL B-lymphocytes dispatch and receive signals to and from the ME, shaping a leukemic niche supporting survival, migration and chemoresistance. As such, CLL B-lymphocyte viability is greatly improved when cocultured with BM-MSCs or monocyte-derived nurse-like cells (NLC) (110, 111). In 2017, Crompot et al. built upon these findings describing that BM-MSC-derived EVs alone could rescue malignant cells from apoptosis and drastically enhance migration and chemoresistance (112). Additionally, uptake of CLL cell-derived sEVs altered the transcriptome of BM-MSCs toward a cancer associated fibroblast (CAF)-like phenotype, promoting CLL leukemic cell survival and disease progression (113). Recent evidence corroborates these findings by demonstrating the miR-146a-dependent downregulation of Ubiquitin Specific Protease 16 (USP16), a repressor of CAF markers [α-smooth muscle actin (α-SMA) and fibroblast activation protein (FAP)] (114). In a 2017 study, Bruns and colleagues illustrated a transformation of healthy donor monocytes into CD14+ HLA-DRlo monocytes belonging to the MDSC population following incubation with CLL-derived EVs (115). Indeed, treatment of monocytes with EVs isolated from CLL cell lines lead to the significant upregulation of IDO1 and downregulation of HLA-DR. Interestingly, these effects were prevented when the monocytes were pretreated with an active vitamin D form (115). Likewise, hy4, a Y RNA highly upregulated in CLL-derived EVs, was shown to induce an immunosuppressive phenotype in monocytes through increased release of C-C motif chemokine ligand (CCL2), CCL4, and interleukin-6 and upregulation of programmed death-ligand 1 (PD-L1) (116). In the same vein, CLL-cells stimulated by T-cell derived signals CD40 and IL-4 secrete sEVs enriched with miR-363, a miR shown to regulate immunomodulatory T-cell receptor CD69 expression (117). These phenotypic changes were complemented by increased migration and immunological signaling. A thorough study by Bottcher et al. investigated the various effect brought upon the T cell population by CLL EV transfer. Activation, proliferation, and survival of T cells are greatly affected by CLL EVs accompanied by marked changes in T cell metabolism and promotion of exhaustion (118). Furthermore, Treg subtypes display a significant induction in the presence of CLL-EVs (118). Taken together with other findings reporting the occurrence of exhausted phenotypes in CD4^+^ and CD8^+^ T cells with decreased cytokine production upon internalization of CD160 containing CLL EVs (119), it is evident that the bidirectional communication between leukemic B cells and T cells is complex. The emerging role of EVs in this crosstalk was further explored by Gargiulo et al. that described the modulatory effects of leukemia microenvironment-derived sEVs (LME-sEVs) on CD8^+^ T-cell function and exhaustion *in vivo* (120). Splenic CD8+ T cells internalizing isolated LME-sEVs display differences in the transcriptome, proteome, and metabolome after 48 hours (120). Gargiulo and colleagues continued with a novel murine model TCL1-RAB27DKO with RAB27 A and B knockout resulting in significantly impaired sEV release (120). They observed delayed CLL progression in TCL1-RAB27KO mice, and when injected into WT C57B/L mice, TCL1-RAB27DKO CLL cells could not carry over CLL progression, suggesting that CLL-sEVs constitute a significant cog in the CLL pathology machinery (120). When presented with the natural cytotoxicity triggering receptor 3 (NCR3)-ligand BAG cochaperone 6 (BAG6), NK cells quickly dispose of tumor cells. However, in CLL, an imbalance of soluble BAG6 in plasma and on the sEV surface compared to CLL B-cell surface may aid in immune evasion due to a lack of surface BAG6 essential for malignant cell targeting (121). The implications of EVs in CLL have been summarized in **Figure 4**.

#### Multiple myeloma

Multiple myeloma (MM) is marked by the uncontrolled proliferation of monoclonal plasma cells that accumulate in the bone marrow (122–124). Malignant plasma cells produce and secrete a monoclonal immunoglobulin protein (M-protein) (122, 123). Low amounts of M-protein in the blood (<3 g/dL) without accompanying symptoms are caused by monoclonal gammopathy of undetermined significance (MGUS), an asymptomatic indolent phase preceding MM (122, 124).

**Figure 5:**
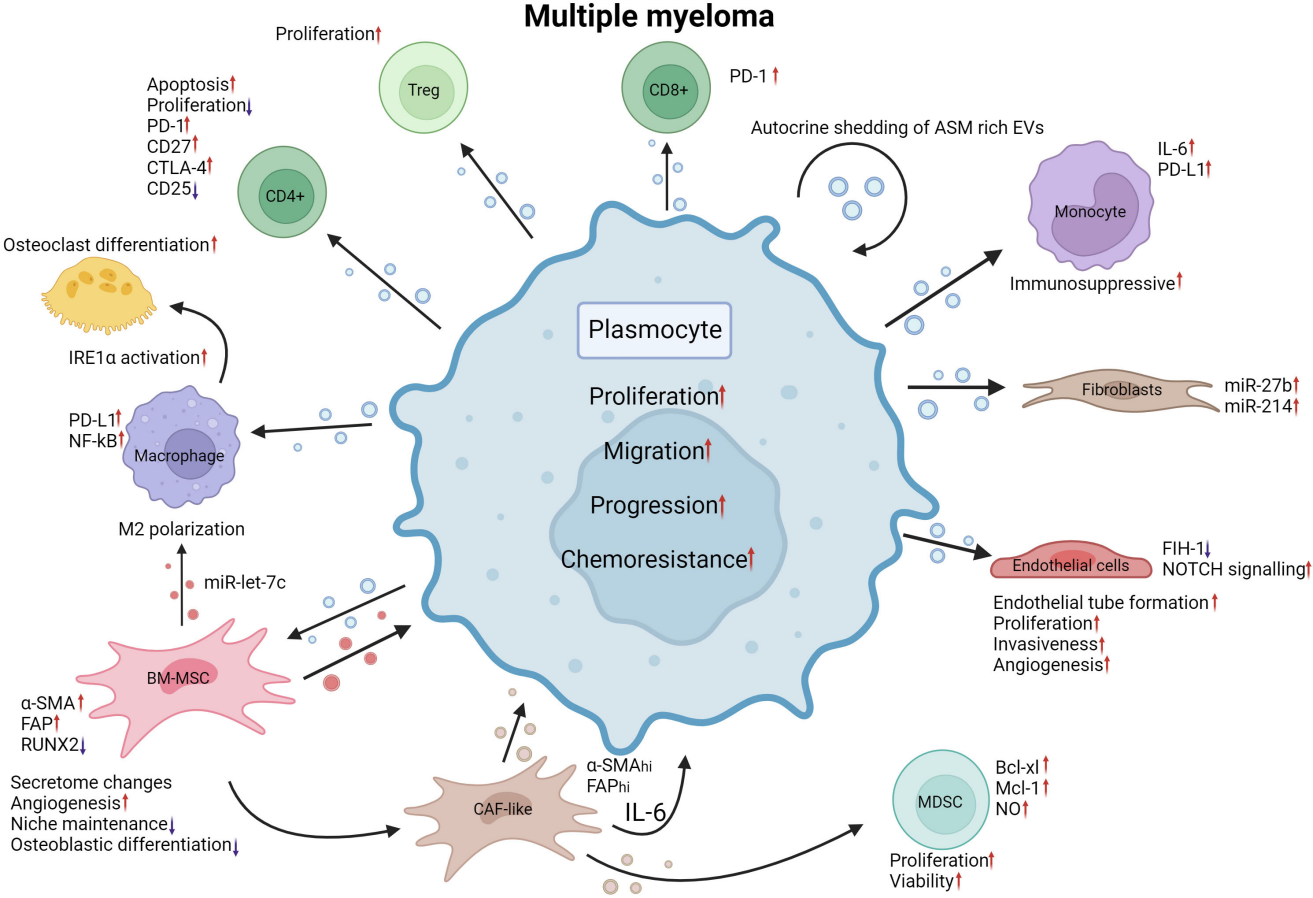
EV mediated crosstalk between MM plasmocytes and their MEs. MM plasmocyte EVs induce Treg proliferation. CD4 + T cell apoptosis is increased, and proliferation is decreased when internalizing MM EVs. PD-L1 expression is increased in macrophages and PD-1 in CD8+ Tcells. NF-kB expression is increased by MM plasmocyte EVs. MM BM-MSC EVs and MM EVs both promote M2 polarization of macrophages by transferring miR-let-7c. IRE1α activation due to plasmocyte EV internalization induces osteoclastic differentiation. MM EVs, when internalized by BM-MSCs, promote the upregulation of α-SMA and FAP while decreasing RUNX2 expression. Secretome changes, increased angiogenesis, decreased niche maintenance and osteoblastic differentiation reprogram BM-MSCs into a CAF-like phenotype, releasing EVs that can promote MDSC expansion through upregulation of BCL-xl, Mcl-1 and increasing NO levels. MM EVs internalized by endothelial cells cause a reduction in FIH-1 expression and an increase in NOTCH signaling. MM EVs promote endothelial tube formation, proliferation, invasiveness, and angiogenesis in endothelial cells. Fibroblasts treated with MM EVs exhibit marked increases in miR-27b and miR-214. Monocytes have increased IL-6 and PD-L1 expression following plasmocyte EV internalization leading to a more immunosuppressive milieu. Autocrine shedding of EVs rich in ASM induces increased chemoresistance in plasmocytes. Together, the alterations brought by MM EVs to the ME promote the proliferation, migration, progression and chemoresistance of MM malignant cells. MM, Multiple myeloma; EV, Extracellular vesicles; PD-1, programmed cell death 1; CD27, cluster of differentiation 27; CD25, cluster of differentiation 25; CTLA-4, Cytotoxic T-Lymphocyte Associated Protein 4; PD-L1, Programmed death ligand 1; NF-kB, Nuclear factor kappa-light-chain-enhancer of activated B cells; IRE1α, endoplasmic reticulum to nucleus signaling 1; α-SMA, Alpha smooth muscle actin; FAP, Fibroblast activation protein alpha; miR, microRNA; RUNX2, RUNX Family Transcription Factor 2; CAF, Cancer associated fibroblast; IL-6, interleukin 6; MDSC, Myeloid derived suppressor cell; BCL-xl, B-cell lymphoma extralarge; Mcl-1, Myeloid Cell Leukemia Sequence 1 (BCL2-Related); NO, nitric oxide; FIH-1, Factor Inhibiting HIF-1; NOTCH, Notch receptor; ASM, acid sphingomyelinase. Created with BioRender.com.

The bone marrow ME is essential for multiple myeloma (MM) progression: the interaction with cells occupying the bone marrow niche can dictate MM cell proliferation, promote angiogenesis, impact bone formation, influence the immune response, impede hematopoiesis and facilitate metastasis (125–129). First, in 2013, Roccaro et al. demonstrated a significant impact of BM-MSC-derived EVs on MM cell proliferation compared to EVs originating from healthy donor (HD)-derived EVs (125). Intriguingly, the content of EVs derived from healthy donor BM-MSCs and MM derived BM-MSCs differed greatly, with distinct proteomic profiles and vastly different miRNomes (125). When exposed to MM BM-MSC-derived EVs, MM cells displayed increased proliferation, tumor growth, greater translation initiation, mitogen-activated protein kinase 1 (MAPK) pathway activation and homing toward the BM niche, indicating that the MM ME alters the BM-MSC secretome (125, 128). Furthermore, the lncRNA LINC00461 is upregulated in MM BM-MSCs and binds miR-15a/16, acting as a sponge for these miRs, resulting in the upregulation of B-cell lymphoma 2 (BCL2), an important antiapoptotic protein normally suppressed by miR-15a/16 (126). Likewise, high levels of miR-10a (130, 131), miR-483 (132) and miR-16 (131) in BM-MSC EVs were linked to increased MM progression, possibly through the regulation of the EPH receptor 8 (EPHA8), insulin-like growth factor 1 receptor/Cyclin D1 (IGF1R/CCND1) axes (131) and tissue inhibitor metalloproteinase 2 (TIMP2) (132). A 2020 report by Debbag and colleagues denotes a high presence of CD49d and CD29 integrins on the surface of BM-MSC EVs corresponding to patient staging. Their absence was marked by a diminished uptake of MM BM-MSC EVs by MM plasmocytes (133). Angiogenesis is essential for tumor growth, and the MM ME was previously shown to promote its inception through soluble factor secretion and EVs (134, 135). MM-derived sEVs have been linked to induced angiogenesis, notably through their impacts on apoptosis- and proliferation-related pathways in BM-MSCs due to their activation of the signal transducer and activator of transcription 3 (STAT3) pathway, resulting in increased cell viability and a significant surge of the number of blood vessels (135). In fact, BM-MSCs of MM patients internalize MM plasmocyte-derived EVs through endocytosis, micropinocytosis and membrane fusion (136), and these EVs are enriched in miR-146a and miR-21, which influence the altered BM-MSCs to secrete cytokines such as IL-6 and TGFβ to establish a ME favorable for MM growth and migration (137). Treatment with endocytosis inhibitors resulted in a marked suppression of the effects brought by the MM-derived EVS (136). Additionally, EVs from MM patients may induce major phenotypic changes in endothelial cells with increased endothelial tube formation, proliferation, and invasiveness by transferring miR-135b (138) and piwi-interacting RNA 823 (139) to endothelial cells (138, 139). miR-135b is overexpressed in MM hypoxia-resistant plasmocyte-derived EVs, and when transferred into endothelial cells, miR-135b was shown to target a factor inhibiting HIF-1, which promotes endothelial tube formation (138). Likewise, piwi-interacting RNA 823 (piRNA823) accumulates in EVs derived from MM patient peripheral blood and is associated with poor outcome. Upon internalization into endothelial cells, piRNA823 promotes proliferation and tube formation and increases the invasiveness of endothelial cells (139). Furthermore, proteomic profiling of MM patient plasma derived EVs revealed enrichment of oncogenic factors linked to cell migration and adhesion (140). NOTCH 2 enrichment within MM-derived EVs may supplement angiogenesis by increasing NOTCH signaling in recipient endothelial cells upon transfer, leading to increased angiogenesis and osteoclastic differentiation (141). Fibroblasts have been described to be influenced by MM cells in a similar manner through the transfer of WW and C2 Domain Containing 2 (WWC2) protein in sEVs (142). When BM fibroblasts capture MM cell sEVs rich in WWC2, *de novo* synthesis of miR-27b and miR-214 occurs, forming a miR profile associated with MGUS to MM progression (142). On the other hand, MM plasmocytes were described to regulate the effects of miRs transferred from fibroblasts through the expression of lncRNAs acting as miR sponges, diminishing miR action and resulting in increased viability (143). Due to the advanced average age of MM patients, BM-MSCs of older patients exhibit a significant reduction in BM niche maintenance (144, 145). Indeed, BM-MSCs originating from young donors displayed a greater antiangiogenic effect than BM-MSCs isolated from older donors (145). For instance, miR-340 expression is diminished in both BM-MSCs and their derived sEVs (144). When artificially overexpressed with miR-340 mimics, BM-MSC-derived sEVs displayed an anti-angiogenic effect through regulation of the hepatocyte growth factor (HGF)/HGF receptor axis (144). Furthermore, the MM microenvironment derived EVs may influence the fate of macrophages by transferring miRs able to drive macrophage M2 polarization through the transfer of overexpressed miR-let-7c, promoting angiogenesis and negatively impacting immune surveillance (146). MM plasmocytes with 13q deletion were also shown to promote M2 macrophage differentiation more strongly than MM plasmocytes without this cytogenetic aberration through the release of miR-16-poor EVs. As a consequence of reduced circulating miR-16, inhibitor of nuclear factor kappa-B kinase subunit alpha/beta (IKKα/β) complex expression of the NF-kB pathway is rescued, resulting in increased MM plasmocyte growth and enhanced M2 macrophage polarization (147). Monocytes undergo PD-L1 and IL-6 upregulation and STAT3 signaling pathway activation when internalizing MM-derived sEVs. This suggests that MM-derived and MM microenvironment derived EVs contribute to the formation of an immunosuppressive ME through the IL-6/STAT3 and HSP72/TLR4/NF-kB axes and miR-let-7c transfer (146, 148). On the other hand, the immunosuppressive aspect of the MM microenvironment is promoted through MDSC support and Treg proliferation (149, 150). EVs isolated from MM BM-MSCs induce MDSC proliferation and increased viability through STAT3 activation, Bcl-xl and Mcl-1 upregulation (135, 149). Similarly, MM cell-derived sEVs were shown to significantly promote apoptosis and inhibit the proliferation of CD4^+^ T-lymphocytes and function of CD8^+^ T-lymphocytes while inducing the proliferation of Tregs (150). Further, Lopes et al. describe a conditioning of the bone marrow *in vivo* upon transfer of MM-derived EVs, leading to PD-1 upregulation in CD4+ T cells, enabling disease progression (151). Taken together, these findings imply narrow ties between the shedding of EVs in MM and the effect they may have to enable a permissive microenvironment facilitating disease progression through the disruption of the immune response and promotion of immunosuppressing populations. Disruption of balanced osteoblastic, osteoclastic and osteocytic activity is the cause of bone lesions commonly occurring in MM patients (152). Myeloma-derived sEVs have been implicated in this disruption, notably by impacting osteoblast and osteoclast differentiation. As such, MM cell-derived sEVs and sEVs isolated from MM patient serum were shown to strongly induce osteoclast activity and differentiation, particularly through the rapid activation of the inositol requiring enzyme 1 (IRE1)/X-box binding protein 1 (XBP1) pathway (153, 154). Murine MM cell-derived sEVs were shown to strongly induce osteoclast activity and differentiation (154). Indeed, the authors uncovered a similar effect between the application of 5TGM1-derived EVs and the presence of MM cells on osteolysis *in vivo*. Dkk-1 transfer through these sEVs led to a marked reduction in RUNX family transcription factor 2 (Runx2), osterix and collagen 1A1 in osteoblasts (154). Further, the transfer of MM-derived exosomes and their contents to BM-MSCs impedes their osteoblastic differentiation through miR-129-5p-dependent inhibition of Transcription factor Sp1, a known positive modulator of osteoblastic differentiation (155). Like other HMs discussed above, EV crosstalk in MM facilitates the chemoresistance of the malignant cells. Firstly, in lenalidomide resistant MM cells, SORT1/LAMP2 dependent enhanced secretion of EVs was observed. Knockdown of Sortilin 1 (SORT1) and Lysosome associated membrane protein (LAMP2) reinstated drug sensitivity in MM cells, indicating the importance of the activity of these genes for lenalidomide resistance (156). Further, by targeting the endocytic pathway essential to BM-MSC-released EV internalization, Tu and colleagues demonstrated through the application of chemical endocytosis inhibitors and endocytosis-associated protein shRNA-mediated knockdown a marked decline in bortezomib (BZ) resistance in MM cells indicating the importance of EV crosstalk in chemoresistance (157). System Xc^-^, a cystine/glutamate antiporter, undergoes noticeable upregulation of its light chain subunit xCT following BZ treatment in both BM-MSCs and MM cells (158, 159). In a 2022 study, Wang and colleagues demonstrated that increased xCT expression following BZ exposure corresponds to increased glutamate and sEV secretion in both BM-MSCs and MM cells (159). MM BM-MSCs may also confer chemoresistance to MM plasmocytes through the release of miR-155-enriched sEVs, as described in MPC-11 cell lines (MM cell line) (160). Acid sphingomyelinase (ASM) is overexpressed in MM cells compared to their healthy counterparts, leading to increased ceramide content. Consequently, when treated with BZ or melphalan, ASM was upregulated further in both MM cells and the sEVs they shed. This high ASM content in sEVs was transferred between MM cells, conferring chemoresistance that could be nullified following ASM inhibitor application (161). The extensive roles of EVs in MM have been summarized in **Figure 5**.

## EVs and their contents as biomarkers

EVs are undeniably of great importance in the crosstalk between the leukemic cells and their microenvironment. Their presence in blood and other bodily fluids allows for a less intrusive liquid biopsy as opposed to classical biopsies for many cancers. As such, several research groups propose the use of EVs as biomarkers for the prognosis of patients in a straightforward and non-invasive analysis. EV have distinct nanomechanical signatures in HMs (162) and as described above, the deregulation of EV cargo is commonplace in HMs (163). Diverging miR content can be revealing for predicting patient outcomes (163). Other cargo components were shown to have easily quantifiable differences such as ncRNA species (164), mRNA (165–167), proteins (168–171) and dsDNA (172) that could guide the treatment strategy, estimate disease progression (173), establishing a prognosis (174), indicate treatment response or even detect minimal residual disease (MRD) (174–181).

The ubiquitous BCR-ABL fusion found in CML has been described to be detected by analyzing CML-derived sEVs (167). Compared to controls, CML sEVs carry this transcript allowing for an easy assessment of BCR-ABL mutation presence (167). Proteome profiling of imatinib resistant CML patient plasma sEVs reveals 279 differentially expressed proteins compared to their imatinib sensitive counterpart (171). Following a bioinformatic analysis, 2 candidate proteins were identified, notably, Ribosomal protein L13 and Ribosomal protein L14, that may serve as imatinib resistance biomarkers in future applications (171).

The prognostic potential of circulating EVs in CLL has long been considered (182). Firstly, changes of miR profile in CLL patient serum is evident, Yeh and colleagues demonstrated a substantial shift in several miRs in CLL patient samples when compared to controls (183). High circulating miR-150 expression in the serum is associated with diminished treatment free survival (TFS) and overall survival (OS) in CLL patients compared to patients with lower amounts (177). Intriguingly, cellular miR-150 levels and circulating miR-150 display opposite prognostic indications, with increased TFS and OS when cellular miR-150 levels were high (177). Additionally, miR-155, shown to be deregulated not only in CLL but in several other HM may serve as a broad biomarker to identify HM occurrences (184). sEV miR quantification was suggested as a predictor of richter syndrome (RS) in CLL patients, a sudden switch to an aggressive form of the disease, with miR-19b serving as a potentially strong indicator of RS transformation and thus a less favorable course (173).

Multiple reports describing the potential of circulating EVs and their content in MM, in particular proteins and sncRNAs as biomarkers have been published. In a 2021 report, Laurenzana et al. demonstrate that MM patients have significantly higher amounts of circulating EVs in their serum compared to healthy controls (185). CD44, a protein involved in the adhesion of MM cells to BM-MSCs, was observed is highly expressed in the EV fraction of corticosteroid resistant MM cell lines and was found to be significantly overexpressed in newly diagnosed MM patients (168). Additionally, high CD44 content detected in patient serum EVs was associated with shorter OS (168). More recently, a high protein/particle ratio, i.e. high protein content within circulating peripheral blood EVs, was associated to immune dysfunction but also significantly lower OS compared to patients with lower protein/particle ratio (169). Following mass spectrometry analysis of peripheral blood EVs of MM patients, the authors uncovered a set of proteins (PDIA3, C4BPA, BTN1A1 and TNFSF13) able to be of use as biomarkers for patient outcomes such as survival, immune dysfunction and treatment response (169). As is the case in other HMs, the level of several microRNAs within serum EVs of MM patients were found to be greatly altered compared to healthy donors and could be linked to patient outcomes. Firstly, seven sEV microRNAs were shown to be decreased throughout the disease progression into MM (healthy controls *vs* monoclonal gammopathy of undetermined significance *vs* MM) leading the authors to believe that this expression-decrease may relate to MM progression and could therefore serve as biomarkers for this phenomenon (186). Moreover, Manier and colleagues observed an association between heightened levels of peripheral blood circulating let-7b and miR-18a in sEVs with PFS and OS of MM patients (187). miR-let-7c, a miR secreted by BM-MSCs was shown to be able to promote the M2 polarization of macrophages, contributing to angiogenesis in the bone marrow ME (146). The authors proposed sEV derived miR-let-7c as a biomarker for MM disease progression (146). Lastly, the profile of sEV-derived circRNA in MM patients was linked to peripheral neuropathy, a complication of MM. Of which, a particular circRNA, namely, chr2:2744228–2,744,407+ was overexpressed in the serum sEVs MM patients and positively correlated with peripheral neuropathy in MM patients (164). MRD monitoring may also be facilitated by the quantification of MM markers on EVs. Recent reports uncovered the potential of liquid biopsies in monitoring MRD by screening EVs isolated in MM remission patients for MM biomarkers such as CD56, CD117, CD27, CD55, CD59, PD-L1 and CD38 (188, 189).

In summary, various HM EVs have been proposed as a powerful non-invasive tool that would enable physicians to generate an early prognosis prediction in a rapid manner. Whether this prognosis relies on the presence of surface markers, proteomic content, dsDNA or sncRNAs, their accuracy remains to be verified. Indeed, a systematic review by Lopez-Santillan et al. disputes the etched in stone viability of EV miR content quantification as non-invasive biomarkers in diffuse large B-cell lymphoma due to high variability between publications (176). Standardization and repeatability of such findings will be crucial for EVs and contents to be of use as biomarkers in the various HMs. This prospect is currently being studied in a clinical trial examining miR and protein deregulation in circulating EVs as tools to predict bone (190). However, no results have yet been reported as per the time of writing to draw any conclusions. A non-exhaustive list of EVs as biomarkers is provided in **Table 1**.

**Table 1:** EV Biomarkers in hematological malignancies.

Biomarker	Factor	HM	Description	Reference
*miR-532*	OS, cellular energy	AML	High expression of plasma sEVs miR-532 in AML patients was associated with a more favorable OS and negatively correlated with cellular energy.	(191)	
*miR-10b*	OS, DFS	AML	Significantly higher miR-10b content was associated with poor OS and DFS in AML.	(191)	
*dsDNA*	Therapy response, CR	AML	Decreasing amounts of sEV dsDNA coincided with better therapy response and complete remission of patients.	(172)	
*Protein content*	Therapy response	AML	Higher protein content at diagnosis in AML sEVs that subsequently decreased following the start of therapy, increased afterwards and finally normalized during long term remission.	(39)	
*TGF-β*	Therapy response	AML	Higher protein content at diagnosis in AML sEVs that subsequently decreased following the start of therapy, increased afterwards and finally normalized during long term remission.	(39)	
*FLT3-ITD mRNA*	Driver mutation	AML	detection of FLT3-ITD mutation in AML derived sEVs.	(192)	
*NPM1*	Driver mutation	AML	detection of NPM1 mutation in AML derived sEVs.	(192)	
*BCR-ABL mRNA*	Driver mutation	CML	CML-derived sEVs carry the BCABL mRNA transcript that can be quantified. Patients in the blast and accelerated phases carry the transcript.	(167)	
*RPL13*	Therapy response	CML	increased RPL13 protein content in MM-derived sEVs was associated with imatinib resistance in CML patients.	(171)	
*RPL14*	Therapy response	CML	increased RPL14 protein content in MM-derived sEVs was associated with imatinib resistance in CML patients.	(171)	
*miR-150*	TFS, OS	CLL	higher circulating miR-150 in the serum was associated with decreased TFS and OS in CLL patients.	(177)	
*miR-155*	Diagnosis	HM	Large variances of miR-155 content across several HM, may therefore serve as a broad biomarker for HM diagnosis.	(184)	
*miR-19b*	Disease progression	CLL	miR-19b associated with the development of CLL into RS.	(173)	
*EV volume*	Disease state	MM	EVs isolated from serum originating from MM patients were more numerous than in healthy sera.	(185)	
*CD44*	OS, Therapy response	MM	CD44 in enriched in EVs derived from corticosteroid resistant MM cell lines, and high CD44 levels in patient serum was associated with reduced OS.	(168)	
*Protein content*	OS, immune dysfunction	MM	A high protein/particle ratio was associated with immune dysfunction and shorter OS compared to patients with a lower ratio.	(168)	
*PDE8B*	Therapy response	MM	PDE8B is upregulated in EVs of VRD resistant MM patients.	(169)	
*PSM8/PSMB8*	Therapy response	MM	PSM8/PSMB8 is upregulated in EVs of VRD resistant MM patients.	(169)	
*miR-20a-5p*	Disease progression	MM	Expression of miR-20a-5p gradually decreased from healthy donors, to smoldering multiple myeloma and multiple myeloma.	(186)	
*miR-103a-3p*	Disease progression	MM	Expression of miR-103a-3p gradually decreased from healthy donors, to smoldering multiple myeloma and multiple myeloma.	(186)	
*miR-4505*	Disease progression	MM	Expression of miR-4505 gradually increased from healthy donors, to smoldering multiple myeloma and multiple myeloma.	(186)	
*miR-140-3p*	Disease progression	MM	Increased expression of miR-140-3p in MM patients compared to SMM patients.	(186)	
*miR-185-5p*	Disease progression	MM	Decreased expression of miR-185-5p in SMM patients compared to healthy controls.	(186)	
*miR-4741*	Disease progression	MM	Increased expression of miR-4741 in SMM and MM patients compared to healthy controls.	(186)	
*miR-20a-5p*	Disease progression	MM	Decreased expression of miR-20a-5p in SMM patients compared to healthy controls.	(186)	
*let-7b*	PFS, OS	MM	Increased levels of EV circulating let-7b associated with better progression-free survival and overall survival compared to low let-7b patients.	(187)	
*miR-18a*	PFS, OS	MM	Increased levels of EV circulating miR-18a is associated with better progression-free survival and overall survival compared to low miR-18a patients.	(187)	
*miR-let-7c*	Disease progression	MM	increased miR-let-7c secretion by MM BM-MSC was associated with M2 polarization, and increased angiogenesis of the bone marrow ME.	(146)	
*chr2:2744228–2,744,407+*	Peripheral neuropathy	MM	increased levels of chr2:2744228–2,744,407+ in MM-derived sEVs positively correlates with peripheral neuropathy incidence in MM patients.	(164)	

## Therapeutic applications

EVs can be shed by most cell types; however, in the context of HMs, malignant cells and crucial cells composing the ME often drive disease progression and interfere with therapy. Exploiting this aspect of the ME may provide targeted therapeutic prospects to counter disease progression. The active use of EVs as carriers inducing apoptosis or repressing the expression of certain genes has been described in several studies in recent years, with promising effects. Induced expression of certain miRs targeting key oncogenes has been adapted to EV delivery to MM cells (181, 193). Targeting of heparinase by miR-1252-5p mimics and elevated sex-determining region Y-related high-mobility-group box transcription factor 4 (SOX4) by miR-335 targeted overexpression resulted in decreased BZ chemoresistance and drastically increased apoptosis, respectively. This demonstrates that key oncogene-targeted regulation through EV transfer lies within the realm of possibility (181, 193). Tumor-derived EVs have been described to carry tumor-associated antigens (TAAs) able to promote tumor-specific immunity by stimulating anticancer responses (194). By downregulating sEV TGF-β1 with lentiviral shRNA, tumor-derived sEVs internalized by dendritic cells (DCs) induced increased expression of surface costimulatory factors and MHC-II complexes in addition to increased inflammatory cytokine secretion, enhancing tumor-specific immunity (195). Similarly, Hu et al. demonstrated lentiviral-mediated induced expression of the costimulatory molecules CD80 and CD86 in a leukemic cell line that led to the release of leukemia-derived EVs with high CD80 and CD86 level that could drastically increase the DC response following internalization (196). DBA/2 mice immunized with leukemia-derived EVs rich in CD80 and CD86 demonstrated dramatically better survival when exposed to L1210 cells than when the mice were immunized with unaltered leukemia-derived EVs (196). Directly blocking the effects of leukemia derived EVs on disease progression and chemoresistance may become a promising avenue to alleviate patient burden. Indeed, by blocking the angiogenic effect of CML-derived EVs with the use of gold nanoparticles, *de novo* vessel formation was steeply reduced compared to the untreated control devoid of CML-derived EVs (197). By inhibiting the endocytic pathway, sEV internalization by MM cells is greatly diminished (157). MM BM-MSC-derived EVs can induce chemoresistance to BZ; however, when the endocytic pathway was inhibited, the apoptotic effect of BZ was enhanced, indicating that targeting sEV internalization by MM cells can be extremely beneficial to improving BZ therapy (157). Finally, the use of EVs as drug delivery systems for improved efficiency in therapy or to reach difficult-to-access areas is key for some treatments. KrasG12D siRNA-loaded exosomes are presently being studied in a clinical trial for pancreatic cancer patients (198). The outcome of this study will lead the way for similar applications in other malignancies. For instance, gp350, an envelope glycoprotein of the Epstein barr virus, is known to bind with great affinity to the CD21 protein on the surface of CLL B-lymphocytes (60). Therefore, Xiu et al. engineered gp350-anchored red blood cell (RBC)-derived EVs that were proven to efficiently bind CLL B lymphocytes (60). They continued by loading fludarabine and doxorubicin into RBC-EVs that were later anchored with gp350, and the EVs had substantial cytotoxic effects on malignant cells (199). Gärtner et al. applied a similar approach by transfecting gp350, CD40L and/or pp65 [an antigen of human cytomegalovirus (CMV)] expression plasmids into HEK293 cells that would later shed gp350+/CD40L+/pp65+ EVs (200).

CAR-T cell derived EVs are a recent avenue being explored in solid cancers (201, 202). Naturally, similar approaches are being explored in hematological malignancies. Haque et al. showed that the treatment of CD19 B-lineage leukemia cell lines with exosomes expressing CD19 Chimeric Antigen Receptor (CAR) induced cytotoxicity and elevated pro-apoptotic genes in CD19-positive leukemia B-cells without inducing cell death in CD19-negative cells (203). Interestingly, the selective characteristic of CAR-T cells is maintained by the EVs they shed, pointing towards their potential for targeted therapy (203).

**Figure 6:**
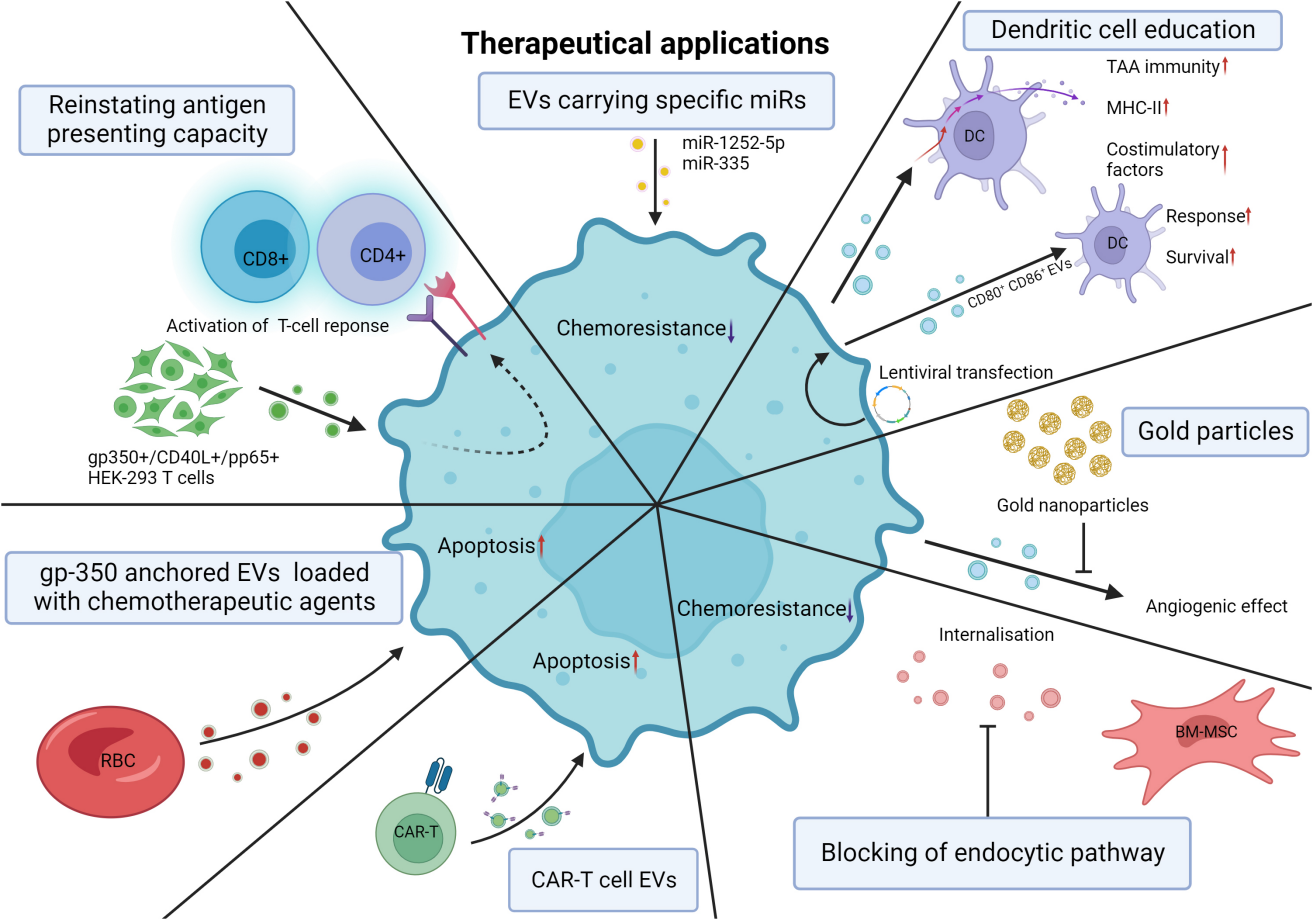
Theoretical therapeutical applications of EVs. Induced expression of miRs adapted to EV delivery. Heparanase targeting by miR-1252-5p mimics and SOX4 targeting by miR-335 overexpression leads to decreased chemoresistance and increased apoptosis of MM cells. Downregulation of sEV TGF-β with lentiviral shRNA causes DCs to internalize tumor-derived sEVs and undergo increased expression of costimulatory factors, MHC-II, and inflammatory cytokines, thereby promoting tumor-specific immunity. Lentiviral transfection of murine leukemic cells results in the release of CD80+ CD86+ EVs increasing the response of DCs and the survival of the mice. *De novo* vessel formation was significantly reduced with the application of gold nanoparticles. Blocking the endocytic pathway of MM cells reduces BM-MSC EV internalization and chemoresistance to bortezomib in MM cells. CAR-T cell derived EVs can selectively target and induce cytotoxicity in leukemic cells. RBC-derived EVs engineered to be anchored with gp350 can be used as drug delivery systems with substantial cytotoxic effects on CLL and BL cells. Transfection of gp350, CD40L and/or pp65 into HEK293T cells results in the shedding of gp350+/CD40L+/pp65+ EVs able to reinstate the antigen presenting capacity of CLL cells and subsequent CD4 and CD8 activation. EV, extracellular vesicle; miR, microRNA; SOX4, sex-determining region Y-related high-mobility-group box transcription factor 4; MM, multiple myeloma; sEV, small extracellular vesicle; TGF-β, transforming growth factor beta; shRNA, short hairpin RNA; DC, dendritic cell; Major histocompatibility complex class II; CD80, Cluster of differentiation 80; CD86, Cluster of differentiation 86; RBC, red blood cell; CAR-T cell, Chimeric antigen receptor T-cell; gp350, glycoprotein 350; CLL, chronic lymphocytic leukemia; CD40L, cluster of differentiation 40 ligand; pp65, human cytomegalovirus tegument protein 65; HEK293T, human embryonic kidney 293 cells. Created with BioRender.com.

By either acting as carriers inducing malignant cell apoptosis or downregulating oncogene expression, EVs have the potential to become invaluable tools to treat HM. However, many challenges remain, and future studies elucidating the applicability of these proposed therapies remain to be performed. Although the limited available studies are promising, much is yet to be studied before the widespread therapeutic use of EVs. The practical applications of EVs in the context of HM have been illustrated in **Figure 6**.

## Discussion

Studies exploring the effects and reach of EVs often encounter problems related to the methods, nomenclature, and reproducibility. For instance, the lack of homogenous protocols throughout the studies referenced in this review, different isolation methods and variations thereof, as well as different media for culturing EV-producing cells, were used. A summary of the methods used in the referenced studies is provided in **Table 2**. Previous studies have demonstrated a lack of consistency, particularly with regards to microRNAs (176, 204). The actual influence that EV-carried miRs have on the microenvironment in hematological malignancies may be overstated as EVs do not all contain microRNAs in a significant manner, neither are these EVs directly fused to the target cell membrane to release their cargo in the cytosol (204). Therefore, more attention should be brought to the other elements within the extracellular vesicles that may participate in the perceived effects often only attributed to microRNAs. Additionally, an argument can be made regarding the use of physiologically relevant doses of EVs in most studies. Compared to the actual microenvironment in these HMs most reports inflate the number of EVs that would meet the cell type of interest. That in turn could lead to observations that would be less likely to occur in physiological conditions. On the other hand, having an insight into the exaggerated effects provided by these EVs can guide us to a better understanding of the inner workings of EV shedding and internalization within the HM microenvironment. Furthermore, inconsistent nomenclature is rife and should be avoided, as the definition of EVs and their subcategories often varies. Therefore, the use of a universally accepted nomenclature, such as MISEV 2018 guidelines (205), will be crucial to avoid misunderstandings. Moving forward, these factors will hamper progress in the field, and efforts should be made to establish proven protocols for consistent and replicable results.

**Table 2:** isolation methods of EVs used in the referred papers when available.

Classification	Malignancy	Isolation method	Reference
Myeloid Neoplasms	AML	Size exclusion chromatography	(39, 40, 63)
		Sequential ultracentrifugation	(39) (43, 41, 46, 48–50, 52, 54–56, 58, 59, 61, 63–68, 195)
		Density gradient ultracentrifugation	(43)
		Precipitation	(44, 51, 53, 57, 28, 62)
		Centrifugation	(45)
		Ultrafiltration	(63, 196)
	CML	Size exclusion chromatography	(73)
		Sequential ultracentrifugation	(70–73, 75–77, 79–82, 171)
		Precipitation	(77, 79, 167, 197)
		Centrifugation	(74)
Lymphoid neoplasms	ALL	Size exclusion chromatography	(94)
		Sequential ultracentrifugation	(95, 96, 98–102)
		Precipitation	(97, 103)
	CLL	Differential ultracentrifugation	(112, 116, 115, 117, 118, 120, 121, 177, 183, 184, 114)
		Density gradient ultracentrifugation	(113, 120, 200)
		Precipitation	(119)
	MM	Sequential ultracentrifugation	(128, 129, 132, 133, 137, 139, 140, 143, (147, 148, 153, 155, 156, 168, 169, 185, 189)
		Density gradient ultracentrifugation	(151, 160)
		Precipitation	(125, 126, 125, 130, 135, 136, 138), (141), (142–146, 149, 150, 154, 157, 159, 161, 164, 181, 186–188)

Key interactions between hematological malignancies and their microenvironment have been proven to be of utmost importance in disease progression. Direct cell-to-cell contact and the release of cyto- and chemokines are known to possess great modular capacity to shape the microenvironment toward a tumorigenic niche. Extracellular vesicles recently became a subject of great interest when their paracrine and autocrine effects in myeloid and lymphoid neoplasms were unraveled. Circulating EVs released by neoplastic cells reach cells within the local ME, enhancing the tumorigenic niche while simultaneously suppressing the immune system and allowing for unhampered proliferation and migration of neoplastic cells. In contrast, cells impacted by the tumor microenvironment that underwent phenotypic changes favoring the disease may shed EVs that can further promote growth and proliferation and, in some cases, enhance chemoresistance, reducing therapeutic efficiency. Naturally, the potency and accessibility of EVs have sparked great interest in academia as tools to improve the detection and treatment of HMs. The non-invasive approach to collect and analyze vesicles compared to more invasive approaches demonstrates the potential of EVs in the diagnosis of HMs and other cancers through routine liquid biopsies. Furthermore, the ability to load therapeutic agents in EVs and targeting malignant cells may prove to be of great aid in cancer that are nonresponsive to therapy. Although many roadblocks lie ahead of EV research, their potential in therapy and diagnosis is limitless.

## Author contributions

DV: Conceptualization, Writing – review & editing, Visualization, Writing – original draft. ND: Conceptualization, Writing – review & editing, Writing – original draft. DB: Writing – review & editing. NM: Writing – review & editing. LL: Conceptualization, Funding acquisition, Supervision, Writing – original draft, Writing – review & editing. BS: Conceptualization, Funding acquisition, Supervision, Writing – review & editing.

## Glossary

**Table:**

ACE	angiotensin-converting enzyme
ADAM10	ADAM Metallopeptidase Domain 10
AKT	Akt Serine/Threonine Kinase
ALL	Acute Lymphoblastic Leukemia
AML	Acute Myeloid Leukemia
ANGPTL2	angiopoietin-like 2
AREG	amphiregulin
ASM	Acid sphingomyelinase
B-ALL	B-Lymphocyte All
BAX	BCL2 associated X, apoptosis regulator
BCL2	B-Cell Lymphoma 2
Bcl-w	B Cell Lymphoma W
Bcl-xl	B Cell Lymphoma Extra Large
BCR	B-Cell Receptor
BM-MSC	Bone Marrow Mesenchymal Stromal Cell
BZ	Bortezomib
CAF	Cancer Associated Fibroblast
CALR	calreticulin
CCL2	C-C Motif Chemokine Ligand 2
CCL4	C-C Motif Chemokine Ligand 4
CCND1	Cyclin D1
CD3d	CD3 Delta Subunit Of T-Cell Receptor Complex
circRNA	Circular Rna
CLL	Chronic Lymphocytic Leukemia
CLL-IPI	Cll-International Prognostic Index
CML	Chronic Myeloid Leukemia
c-MYB	Myb Proto-Oncogene
CXCL12	C-X-C motif chemokine ligand 12
CXCR4	C-X-C chemokine receptor type 4
DC	Dendritic Cell
Dkk1	dickkopf WNT signaling pathway inhibitor 1
DPP4	dipeptidyl-peptidase 4
dsDNA	Double Stranded Dna
EGFR	Epidermal Growth Factor
EHD4	EH Domain Containing 4
EPHA8	EPH receptor 8
ERK	Extracellular Regulated Kinase
ESCRT	Endosomal Sorting Complex Required For Transport
EVs	Extracellular Vesicles
EZH2	Enhancer Of Zeste 2 Polycomb Repressive Complex 2 Subunit
FAP	Fibroblast Activation Protein
FAS	Fas Cell Surface Death Receptor
FASL	Fas Cell Surface Death Receptor Ligand
FGF2	fibroblast growth factor 2
FoxP3	Forkhead Box P3
gDNA	Genomic Dna
GP96	Glycoprotein 96
GRB10	Growth Factor Receptor Bound Protein 10
GSK-3β	and glycogen synthase kinase-3 beta
HD	Healthy Donor
HLA-DR	major histocompatibility complex, class II, DR alpha
HM	Hematological Malignancy
HMGCR	3-Hydroxy-3-Methyl-Glutaryl Coenzyme A Reductase
HPC	Hematopoietic Progenitor Cell
HSC	Hematopoietic Stem Cell
HSP70	Heat shock protein 70
HSPC	Hematopoietic Stem And Progenitor Cell
HUVEC	Human Umbilical Vein Endothelial Cells
IDO1	Indolamine-2,3-Dioxygenase
IFN-γ	Interferon- γ
IGF1R	Insulin Like Growth Factor 1 Receptor
IKK	Inhibitor Of Kappa B Kinase
IL-10	Interleukin-10
IL-8	Interleukin - 8
ILV	Intraluminal Vesicles
iNOS	Nitric Oxide Synthase
IRE1	inositol requiring enzyme 1
JAG1	Jagged canonical Notch ligand 1
JAK2	janus kinase 2
KRAS	Kirsten Rat Sarcoma Viral Oncogene Homolog
LAMP2	Lysosome associated membrane protein 1
LDOC1	Leucine Zipper Downregulated In Cancer
LME-sEVs	leukemia microenvironment-derived sEVs
LPS	lipopolysaccharides
LRIG1	Leucine Rich Repeats And Immunoglobulin Like Domains 1
LSC	Leukemic Stem Cell
m/lEVs	Medium to Large Extracellular Vesicles
MAPK	Mitogen Activated Protein Kinase 1
mDNA	Mitochondrial Dna
MDSC	Myeloid Derived Suppressor Cells
ME	Microenvironment
MGUS	Monoclonal Gammopathy Of Undetermined Significance
MHC-II	Major Histocompatibility Complex
miR	microRNA
MLCK	myosin light-chain kinase
MM	Multiple Myeloma
MMP9	matrix metalloproteinase 9
MPL	MPL proto-oncogene
MPN	myeloproliferative neoplasms
M-protein	Monoclonal Immunoglobulin Protein
MSC	Mesenchymal Stromal Cell
mTOR-S6	mammalian Target of Rapamycin – ribosomal s6 kinase
MVB	Multivesicular Body
NCR3	triggering receptor 3
ncRNA	Non-coding RNA
NFATc3	Nuclear Factor of Activated T Cells 3
NF-kB	Nuclear Factor Kappa B
NK	Natural Killer Cell
NKG2D	Natural Killer Group 2D
NLC	Nurse-Like Cells
NO	Nitric Oxide
NOTCH1	Notch receptor 1
NQ01	Nad(P)H Quinone Dehydrogenase 1
PBMC	Peripheral Blood Mononuclear Cells
PD-1	Programmed cell death protein 1
PD-L1	Programmed Death-Ligand1
PERK	PRKR-like endoplasmic reticulum kinase
piRNA	Piwi-Interacting Rna
PLEKHM1	Pleckstrin homology domain family M member 1
PMF	Primary myelofibrosis
PS	Phasphatidyl Serine
RAB	Ras Associated Binding
ROS	Reactive Oxygen Species
Runx2	Runt-Related Gene 2
SCF	Scf complex
SDF1	Stromal cell-derived factor 1
sEVs	Small Extracellular Vesicles
SNAI1	Snail family transcriptional repressor 1
SNARE	Snap Receptor
snRNA	Small Nuclear Rna
SORT1	Sortilin 1
SOX4	Sex-Determining Region Y-Related High-Mobility-Group Box Transcription Factor 4
ssDNA	Single Stranded Dna
STAT3	Signal Transducer And Activator Of Transcription 3
S100A4	S100 calcium binding protein
TAAs	Tumor Associated Antigens
T-ALL	T-Lymphocyte All
TGF-β1	Transforming Growth Factor-β1
TIMP2	tissue inhibitor metalloproteinase 2
TKIs	tyrosine kinase inhibitors
TLR2	Toll-Like Receptor 2
TNF-α	Tumor Necrosis Factor – Α
Tregs	Regulatory T-Cells
TRIM28	Tripartite Motif Containing 28
TSG-101	Tumor Susceptibility 101
TSPAN3	Tetraspannin 3
USP16	Ubiquitin specific protease 16
VEGF	Vascular Endothelial Growth Factor
WNT5a	Wnt Family Member 5A
WWC2	Ww And C2 Domain Containing 2
XBP1	X-box binding protein 1
α-SMA	Α-Smooth Muscle Actin
